# OX40 signaling in cancer immunotherapy: mechanisms of action, translational applications, and therapeutic perspectives

**DOI:** 10.3389/fimmu.2026.1724756

**Published:** 2026-02-09

**Authors:** Yuehua Luo, Jing Li, Le Li, Bingbing Qin, Ruisheng Zhou, Ying Tang

**Affiliations:** 1 Guangzhou University of Chinese Medicine, Guangzhou, Guangdong, China; 2 Science and Technology Innovation Center, Guangzhou University of Chinese Medicine, Guangzhou, Guangdong, China

**Keywords:** cancer immunotherapy, combination therapy, costimulatory signaling, OX40, tumor microenvironment

## Abstract

OX40 (CD134/TNFRSF4), a costimulatory receptor of the TNF receptor superfamily ((TNFRSF), has emerged as a compelling immuno-oncology target given its capacity to amplify T-cell activation, sustain effector and memory responses, and remodel the tumor microenvironment (TME). This review provides a comprehensive synthesis of OX40 biology from molecular architecture to pathway-specific signaling programs, emphasizing its distinct yet interconnected roles across CD4^+^ T-cell subsets, CD8^+^ T cells, T follicular helper cells, and regulatory T cells (Tregs). We further summarize the landscape of OX40 expression across major solid tumors, highlighting its heterogeneous prognostic significance and the immune-contextual factors that determine therapeutic responsiveness. Although early-phase clinical studies of OX40 agonists have demonstrated favorable tolerability and robust pharmacodynamic activation, their antitumor efficacy either as monotherapy or in combination with PD-1/PD-L1 or CTLA-4 inhibitors has remained modest. Mechanistic barriers such as transient OX40 expression kinetics, Treg counteractivation, metabolic suppression, and insufficient FcγR-mediated crosslinking likely underlie this translational gap. Emerging bispecific antibody platforms and OX40-integrated combinatorial regimens offer renewed opportunities to overcome these limitations by enabling spatially controlled receptor clustering, TME-selective activation, and multi-pathway synergy. Future translational success will require refined dosing strategies, optimized antibody engineering, biomarker-guided patient selection, and integrated approaches that align OX40 activation with favorable immune dynamics in the TME.

## 1 Introduction

Immunotherapy has revolutionized cancer treatment and is now recognized as one of the most promising therapeutic modalities. Major strategies include immune checkpoint inhibitors, CAR-T cell therapy, and cancer vaccines (1). These approaches enhance the immune system’s capacity to selectively eliminate tumor cells while generally maintaining acceptable safety profiles. Despite the success of immune checkpoint blockade (ICB), its efficacy varies considerably across tumor types and patient populations (2, 3). Several clinical trials of ICB have reported low response rates, limited clinical benefit, or the emergence of immune resistance, which are frequently associated with T-cell exhaustion and an TME enriched not only for Tregs but also for other suppressive subsets such as tumor-associated macrophages (TAMs) and myeloid-derived suppressor cells, as well as tumor cells that express inhibitory ligands and secrete immunosuppressive mediators including TGF-β, IL-10 and IDO (4–9). Overcoming these limitations, particularly in relapsed or refractory cancers, remains a critical priority in current research.

Recent research has increasingly focused on costimulatory molecules within the TNFRSF, including OX40, 4-1BB, and GITR for their capacity to enhance T cell activation (10, 11). OX40 engagement with its ligand OX40L triggers downstream signaling pathways that promote T cell proliferation, survival, and effector function, thereby amplifying antitumor immunity (12, 13). However, reported effects of OX40 signaling appear heterogeneous: while many studies show that OX40 enhances effector T-cell responses and restrains Treg-mediated suppression, other reports suggest that OX40 may also support Treg expansion or activation under certain contexts, reflecting unresolved discrepancies in the field (14–18). Given these conflicting findings, it is important to briefly introduce—but not overinterpret—the current evidence base in the Introduction and to provide a detailed mechanistic evaluation in subsequent sections.

Early-phase clinical trials further indicate that these agents are well tolerated and capable of activating effector T cells while reprogramming TME (19–21). This review systematically examines the immunoregulatory mechanisms of OX40, with particular emphasis on its effects on T cell subsets and the TME, and summarizes recent progress in the development of OX40 agonists.

## 2 Molecular and cellular characteristics of OX40

OX40, a member of Tumor necrosis factor receptor superfamily (TNFRSF), was first identified in 1987 on activated rat CD4^+^ T cells and subsequently characterized as a T cell costimulatory molecule (22–24). It is absent on naïve T cells but is induced upon activation in CD4^+^ T cell subsets as well as in CD8^+^ T cells (25). The expression of OX40 is not observed on naïve T cells but is induced in a temporally regulated manner following antigen-specific TCR engagement during early T cell activation (26). Low-level expression has also been detected in neutrophils, NK cells, NKT cells, and certain non-lymphoid cells (27). Functionally, OX40 regulates T cell survival, expansion, and memory formation, thereby playing key roles in models of infection, autoimmunity, and cancer (28). Clinical studies further support its potential as an antitumor therapeutic target (29, 30). Although low-level OX40 expression has been reported in other T-cell subsets, extensive immunophenotyping demonstrates that OX40 is predominantly expressed by CD4^+^ T cells, with the highest levels consistently observed on Tregs, particularly in cancer settings (31).

Accumulating evidence indicates that OX40 expression is regulated in a hierarchical manner. TCR signaling is indispensable for the initial induction of OX40, as OX40 is not detectably expressed on naïve T cells and is upregulated only following antigen-specific activation (32–34). In contrast, inflammatory cytokines do not independently induce *de novo* OX40 expression but instead function as secondary modulators that reinforce or prolong OX40 expression in already activated CD4^+^ T cells (35). Among these, common γ-chain cytokines such as IL-2, IL-7, and IL-15 have been reported to enhance the magnitude or persistence of OX40 expression by sustaining T cell activation states and engaging downstream STAT5-dependent transcriptional programs (36). Through this mechanism, cytokine signaling amplifies OX40-mediated costimulation without bypassing the requirement for TCR engagement, thereby integrating antigen specificity with contextual immune cues.

OX40L, the specific ligand for OX40 and a member of the TNF ligand superfamily (TNFSF), was initially identified based on its similarity to human glycoprotein 34, a protein expressed on T cells whose expression is regulated by the tax gene of the retrovirus human T-cell lymphotropic virus type 1 (HTLV-1) (37). OX40L its major sources are activated professional antigen-presenting cells (APCs), including dendritic cells (DCs), macrophages, and B cells (38–41), but it is also present on non-immune cells, including smooth muscle and endothelial cells (42, 43). Notably, recent studies have identified group 2 innate lymphoid cells (ILC2s) as an inducible source of OX40L during allergic inflammation, contributing critically to Th2 polarization (44, 45). OX40L expression can be detected on activated CD4^+^ and CD8^+^ T cells, with IL-12 significantly upregulating its expression, particularly in CD4^+^ T cells, where levels exceed those in CD8^+^ T cells (46, 47). Beyond T cell regulation, OX40/OX40L signaling also promotes dendritic cell differentiation and maturation. CD40 stimulation (e.g., via soluble CD40L) induces OX40L expression on immature monocyte-derived DCs, and subsequent OX40L ligation upregulates CD80, CD86, CD54, and CD40 while markedly enhancing the secretion of IL-4, IL-6, IL-12, TNF-α, and IL-1β by approximately 4- to 35-fold (48, 49).

**Figure 1:**
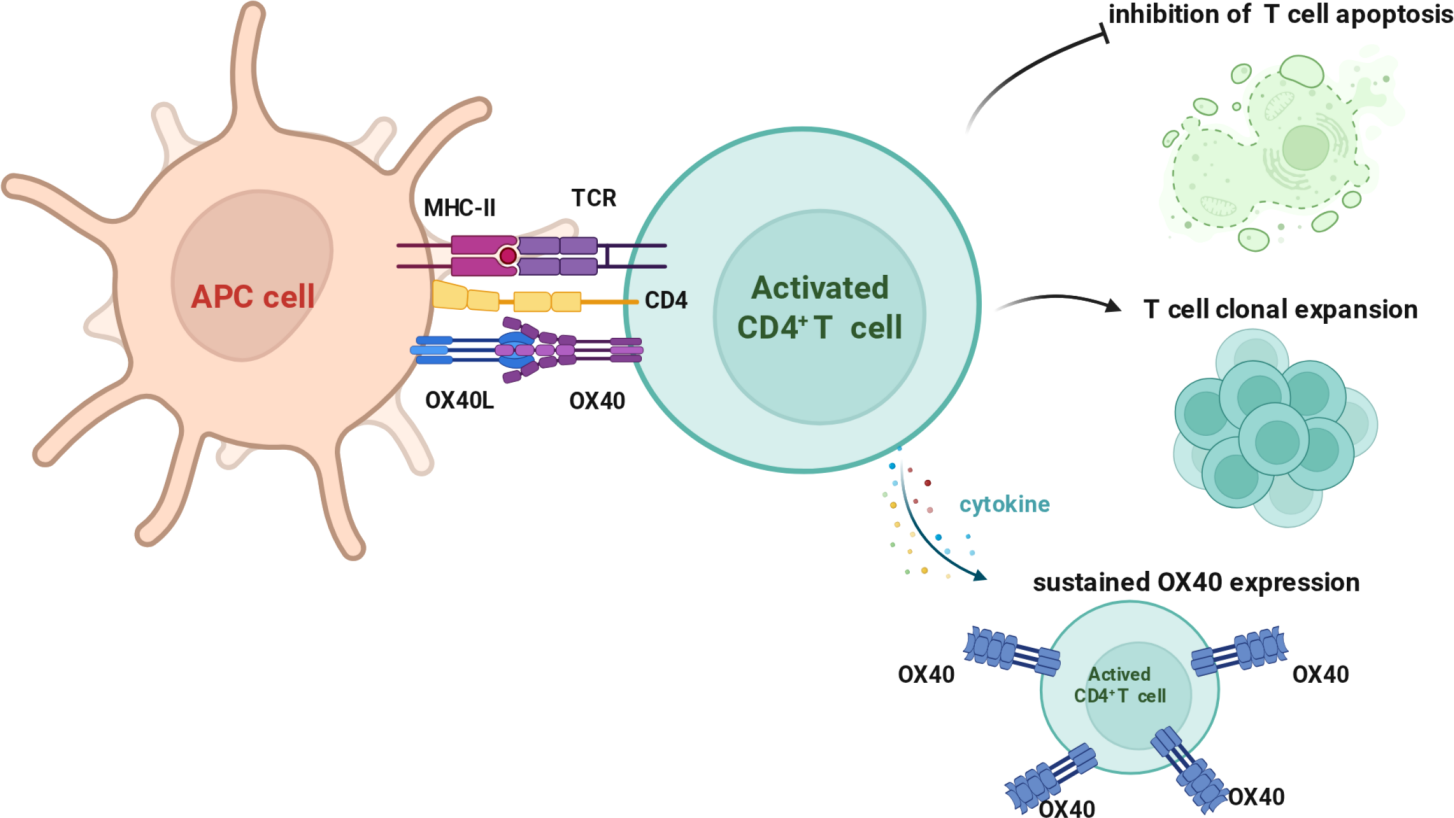
Molecular and cellular characteristics of OX40/OX40L. Upon antigen presentation, engagement of the T cell receptor (TCR) and CD4 coreceptor with peptide–MHC class II complexes on APCs induces activation of CD4^+^ T cells and transient upregulation of the costimulatory receptor OX40. Concurrent interaction between OX40 and its ligand OX40L expressed on APCs provides essential costimulatory signals that inhibit activation-induced T cell apoptosis and promote clonal expansion. Cytokines produced in the activated immune microenvironment further reinforce this process by sustaining OX40 expression on CD4^+^ T cells, thereby stabilizing pro-survival and proliferative signaling programs. Sustained OX40 expression enables prolonged costimulatory signaling, supporting continued T cell expansion and functional persistence.

Structurally, OX40 is a homotrimeric type I transmembrane receptor, and its ligand OX40L also forms a homotrimer, a conserved feature across the TNFR-TNFSF family (50). The fundamental unit of the receptor-ligand complex is a 3:3 hexameric assembly, consisting of one ligand trimer and three receptor molecules (**Figure 1**). This geometric arrangement is a conserved feature shared by multiple TNF-receptor complexes and is widely regarded as the basic structural unit for signal transduction (51). Productive signaling requires ligand-induced receptor clustering rather than simple receptor-ligand engagement (52, 53). Membrane-bound OX40L induces higher-order receptor clustering, thereby markedly enhancing downstream signaling, whereas soluble OX40L generally exhibits weaker activity unless additional cross-linking or oligomerization occurs. This observation reflects a fundamental structural principle of the TNFR superfamily, whereby receptor clustering is indispensable for effective signal transduction (54, 55). This structural principle dictates that antibodies targeting different extracellular cysteine-rich domains (CRDs) of OX40 yield distinct agonistic outcomes: the agonistic efficacy of anti-OX40 antibodies is highly dependent on the specific CRD region they bind. Antibodies directed against the membrane-proximal CRD4 exhibit the strongest receptor clustering capability, thereby generating the most potent T-cell co-stimulatory signals; those targeting CRD2-CRD3, by occupying the ligand-binding region, typically function as ligand blockers and require FcγR-mediated crosslinking to achieve moderate agonistic effects; whereas antibodies binding the outermost CRD1 demonstrate minimal or absent agonistic activity due to their inability to form effective transmembrane clustering. Overall, the CRD location determines whether an antibody can induce optimal trimeric clustering near the membrane, which directly governs its agonistic potency (56, 57). Consequently, the targeted CRD dictates receptor clustering efficiency, FcγR dependency, and signaling profiles, thereby providing a critical structural foundation for the rational design of OX40-targeted therapies (58–60).

OX40 signaling is mediated through adaptor proteins of the TRAF family. The cytoplasmic TRAF-binding motif (aa 256–263, GGSFRTPI) recruits TRAF2, TRAF3, and TRAF5 (61, 62). TRAF2 and TRAF3 activate the canonical (NF-κB1) and noncanonical (NF-κB2) pathways, respectively, while TRAF6 contributes to late-phase NF-κB signaling (61, 62). Upon OX40–OX40L engagement, the OX40-TRAF2 complex translocates to membrane lipid rafts, where it assembles with PI3K and its downstream effector PKB/Akt to form a signaling complex (63, 64), This spatial redistribution is critical for the recruitment and activation of the PI3K/Akt cascade and requires both intact TRAF2 function and proper localization of OX40 within lipid rafts. Notably, TRAF2 knockdown not only markedly impairs canonical NF-κB activation but also disrupts PI3K and Akt recruitment, underscoring the non-redundant and spatiotemporally precise role of TRAF family adaptors in OX40 signal transduction (65, 66). In addition to these NF-κB and PI3K/Akt pathways, OX40 costimulation has been shown to potentiate TCR-mediated NFAT activation, leading to enhanced IL-2 transcription, improved T-cell survival, and augmented proliferative capacity (67, 68). Collectively, these cascades regulate T cell survival, proliferation, and the magnitude of immune responses.

OX40 (TNFRSF4) signals through the TRAF2/5 and PI3K–Akt pathways to regulate a core set of survival, differentiation, and immunoregulatory genes. Upon OX40 engagement, Bcl-2 and Bcl-xL are rapidly upregulated, supporting the sustained survival of activated CD4^+^ T cells, whereas OX40 deficiency leads to their decline and subsequent apoptosis (32). OX40 also drives persistent expression of Survivin (Birc5) via PI3K/PKB, enabling clonal expansion and rescuing proliferation in costimulation-deficient T cells (69). In CD4^+^ T cell-derived cDNTs, OX40 further modulates Bcl-2, Bcl-xL, Survivin, and BCL2L11 (Bim) to maintain proliferation and antiapoptotic capacity, with IL-2 enhancing this program through OX40 upregulation (70). In Th2 differentiation, OX40 promotes early IL-4 transcription and GATA-3 nuclear accumulation, thereby initiating IL-4-dependent and -independent Th2 programs (71). Conversely, OX40/OX40L signaling suppresses IL-10 expression and Tr1 induction pathways, shifting immunity away from tolerance (72). Overall, the OX40 downstream network—centered on Bcl-2 family genes, survivin, IL-4/GATA-3, and IL-10—defines its role in controlling T-cell survival, polarization, and clonal expansion. Building on the molecular pathways and functional modules described above, we further categorized the key genes induced or modulated by OX40 and summarized them systematically in **Table 1**.

**Table 1:** Representative genes induced or modulated downstream of OX40 signaling.

Functional module of OX40 Signaling	OX40-Induced or modulated Genes	Primary cellular function	Mechanistic link to OX40 pathway	Refrence
T-cell survival & anti-apoptosis	BCL2, BCL2L1 (Bcl-xL)	Enhance anti-apoptotic capacity and promote long-term cell survival	Upregulated through NF-κB and PI3K–Akt–dependent transcriptional activation	(32)
	BIRC5 (Survivin)	Support cell-cycle progression and clonal expansion	Sustained expression driven by OX40-mediated activation of the PI3K–Akt pathway	(69)
	Upregulation of BCL2/BCL-xL and downregulation of BIM	Balance prosurvival and proapoptotic programs	Modulated through activation of the noncanonical NF-κB pathway	(70)
T-cell activation & cytokine production	IL2, IL4	Promote T-cell proliferation and survival, Drive Th2 differentiation	IL-2 transcription enhanced by cooperative NFAT and NF-κB activation, Transcription promoted by the OX40–PI3K–NFAT axis	(71)
	IFNG (IFN-γ)	Enhance Th1/CTL antitumor effector activity	Increased through reduced Treg suppression and strengthened effector differentiation	(73)
Regulatory T-cell modulation	FOXP3 (downregulated)	Reduce Treg suppressive activity	OX40 signaling destabilizes Foxp3 expression and inhibits induced Treg formation	(73)
	IL10 (functional modulation)	IL-10 limits effector T-cell activation and maintains immune tolerance.	OX40L signaling blocks the differentiation of IL-10–producing Tr1 cells and suppresses IL-10 production in existing Tr1 cells,	(72)

Notably, the transcriptional program downstream of OX40 aligns with a refined conceptual framework that distinguishes TNFRSF-mediated co-stimulation from the classical IgSF-derived “signal 2.” Unlike CD28-family receptors, which are constitutively expressed on naïve T cells and primarily initiate early activation, several activation-inducible TNFRSF members—including OX40, 4-1BB, and GITR—provide delayed but sustained support to effector T cells by upregulating anti-apoptotic mediators such as BCL-2, Bcl-xL, and survivin (32, 69). These observations support a revised definition of TNFRSF-derived inputs as a distinct “signal 4” whose principal function is to prolong and stabilize T-cell responses rather than initiate priming (74, 75).

## 3 Expression and functional mechanisms of OX40 in T cells

### 3.1 Expression and regulation of OX40

OX40 is a tightly controlled, activation-dependent receptor whose induction reflects the strength, duration, and costimulatory context of TCR engagement. In both human CD4^+^ and CD8^+^ T cells, OX40 is rapidly but transiently upregulated following TCR stimulation, with a more sustained and higher-magnitude response in the CD4^+^ compartment, particularly within memory CD4^+^ T cells and Tregs (76–81). In tumors, Tregs retain the most robust OX40 expression, consistent with their activated phenotype in the TME.

OX40 induction requires more than TCR signaling alone and is tightly coupled to the activation status of T cells. Unlike CD28, which is constitutively expressed on naïve T cells and provides essential costimulatory signals during the priming phase, OX40 is an inducible costimulatory receptor whose expression is upregulated following antigen-specific activation. CD28-mediated costimulation significantly amplifies OX40 transcription in activated CD4^+^ T cells, while CD40–CD40L interactions further enhance OX40 expression by reinforcing APC–T cell communication (32, 82, 83). These integrated signals ensure that OX40 functions as a context-dependent checkpoint of T cell activation, accessible only under conditions of strong antigenic stimulation and appropriate costimulatory cues. During thymic development, OX40 expression can also be detected in subsets of thymocytes undergoing positive selection, reflecting a potential role in fine-tuning TCR repertoire selection (84). Outside the conventional T cell lineage, OX40 expression is generally low but has been reported to be inducible in a context-dependent manner in NKT cells, NK cells, and neutrophils (11, 82, 85).

### 3.2 The role of OX40 in CD4^+^ T cells

The OX40-OX40L interaction enhances T-cell survival, proliferation, cytotoxicity, and memory formation, while simultaneously mitigating the immunosuppressive activity of Tregs. Together, these effects promote broad and sustained T-cell activation (86). A single administration of an OX40 agonist antibody is sufficient to disrupt peripheral tolerance in CD4^+^ T cells, enabling their expansion and restoring effector activity (87). Mechanistically, OX40 signaling amplifies effector functions by upregulating key cytokines such as IL-2, IL-4, IL-5, and IFN-γ (86).

The role of OX40 is particularly relevant to memory T-cell pool formation. Although naïve CD8^+^ T cells can expand up to ~50,000-fold after TCR stimulation, 90–95% of effectors undergo apoptosis at the contraction phase, with only 5–10% persisting as long-lived memory cells (88). OX40 signaling helps counteract this attrition by delivering critical survival signals that favor memory cell development.

**Figure 2:**
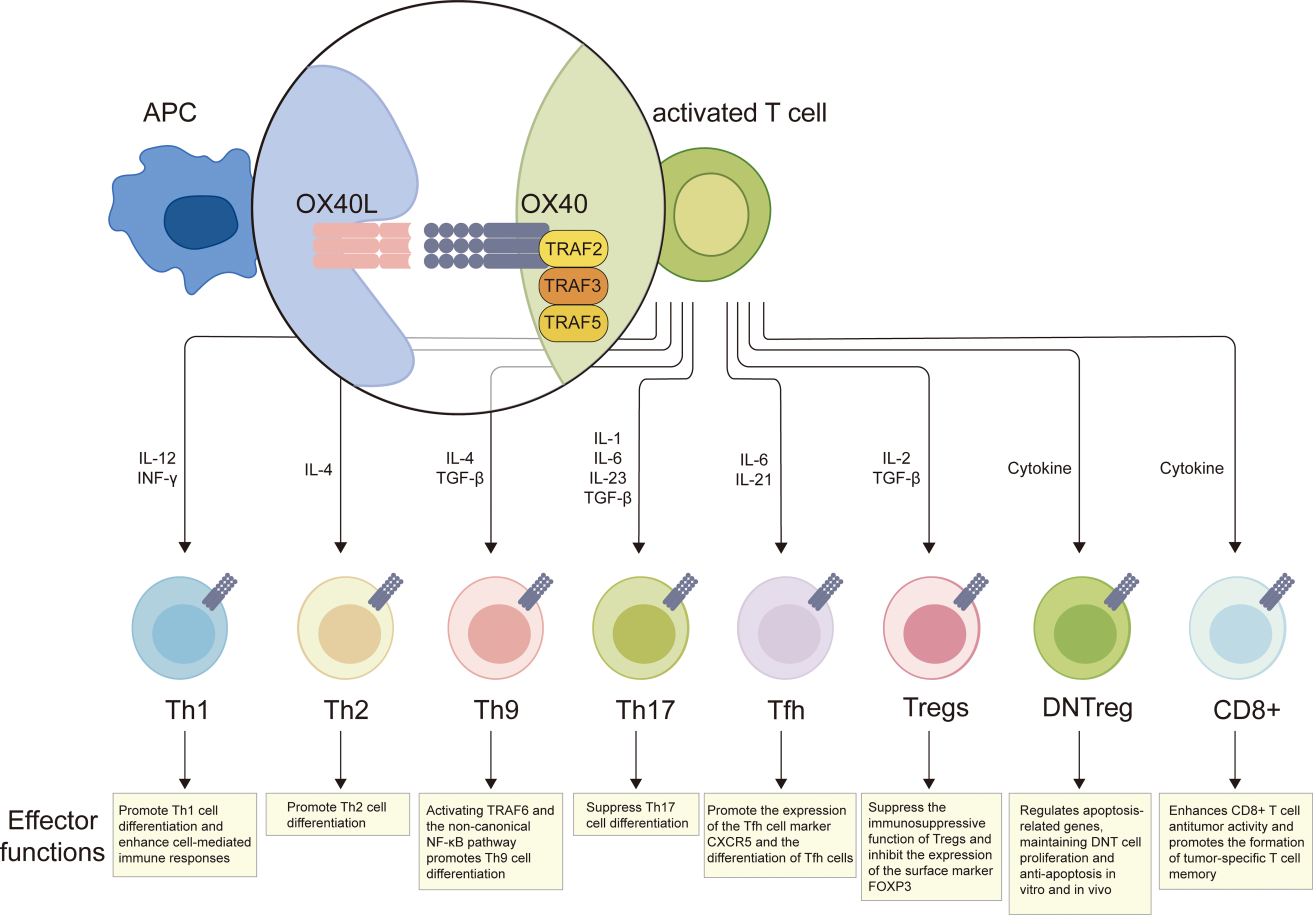
The role of OX40 signaling in the differentiation and functional regulation of T cell subsets. OX40 engagement by OX40L activates downstream signaling via TRAF2, TRAF3, and TRAF5 adaptor proteins. This cascade modulates polarization and effector functions in CD4^+^ and CD8^+^ T cell subsets. OX40 promotes Th1 differentiation (enhancing cell-mediated immunity), facilitates Th2/Th9 differentiation (Th9 induction requires TRAF6 and non-canonical NF-κB), and suppresses Th17 differentiation. It upregulates T follicular helper (Tfh) cell markers and supports Tfh development. Conversely, OX40 signaling inhibits Treg immunosuppression by downregulating FOXP3. Additionally, OX40 supports the survival and anti-apoptotic function of double-negative regulatory (DNT), and boosts CD8^+^ T cell-mediated antitumor activity and the formation of tumor-specific memory T cells. Created with BioRender.com.

Importantly, the regulatory functions of OX40 are stage-specific. During the early activation phase (2–3 days post TCR engagement), OX40-deficient T cells retain the ability to proliferate and differentiate into effector cells, suggesting minimal involvement in initiation (89). In contrast, during the effector maintenance phase (days 12-13), OX40-deficient T cells exhibit markedly reduced survival, underscoring the necessity of OX40 signaling for sustaining effector populations (46). Comparative studies further demonstrate that CD28 signaling is indispensable for robust IL-2 secretion during early activation, whereas OX40 deficiency results only in moderate reductions in IL-2 production (90). Thus, CD28 primarily governs the initiation of T cell responses, while OX40 plays a dominant role in maintaining effector function and survival. Collectively, OX40 signaling exerts critical control over T-cell subset differentiation and long-term immune function (**Figure 2**). Notably, the impact of OX40 is not uniform across T-cell lineages; its regulatory roles diverge in CD4^+^ T cells, CD8^+^ T cells, and Tregs, highlighting the importance of examining OX40 signaling within distinct T cell subsets.

#### 3.2.1 OX40 costimulation in effector and memory CD4^+^ T cells

OX40 is a pivotal costimulatory receptor that plays a central role in enhancing CD4^+^ T-cell activation, effector differentiation, and memory formation, thereby contributing to the establishment of durable antitumor immune responses (29, 32, 33). Upon TCR engagement, OX40 signaling promotes T-cell survival, proliferation, and cytokine secretion, providing essential signals that sustain effector T-cell function under conditions of chronic antigenic stimulation, such as within the TME (32).

Upon engagement of OX40 by OX40L or agonistic antibodies, the cytoplasmic tail of OX40 recruits TRAF2 and TRAF5, whereas TRAF3 predominantly serves as a negative regulator (62). These adaptor molecules initiate the canonical NF-κB (NF-κB1) signaling cascade via TRAF2/5, resulting in IκBα phosphorylation and degradation, nuclear translocation of p50–RelA, and consequently enhanced proliferation and survival of effector CD4^+^ T cells (91). TRAF3 acts as a negative regulator by suppressing OX40-TRAF2-mediated NF-κB activation through both its N- and C-terminal domains, while having no impact on the NIK–IKKα cascade, suggesting that TRAF3 negatively regulates signaling between TRAF2 and NIK (64, 92).

Multiple studies indicate that OX40 signaling extends beyond the canonical NF-κB pathway and critically activates the PI3K-AKT axis through a TRAF2-dependent mechanism (65, 93). Following ligand engagement, OX40 translocates into lipid rafts where it forms a TRAF2-anchored signaling complex that recruits PI3K and AKT, thereby sustaining TCR-dependent AKT phosphorylation (69, 94). This persistent OX40 costimulation maintains PI3K-PKB (AKT) activity, driving clonal expansion and promoting long-term T-cell survival. A hallmark of this pathway is the sustained expression of Survivin, which facilitates S-phase progression, supports cell division, and protects activated T cells from apoptosis, thereby restoring proliferative capacity and *in vivo* expansion even in the absence of continued costimulation. In contrast, antigen-stimulated CD4^+^ T cells lacking OX40 fail to sustain PKB activity and rapidly undergo apoptosis (69). Importantly, enforced PKB activation rescues the survival defects of OX40-deficient T cells, prevents downregulation of anti-apoptotic factors, and reinstates inflammatory responses. Collectively, these findings indicate that persistent Survivin expression coupled with periodic PKB signaling downstream of OX40 cooperatively governs effector T-cell proliferation, survival, and functional persistence, thereby supporting durable immune responses (94).

In summary, OX40 enhances CD4^+^ T-cell proliferation, survival, and effector function, and sustains long-term immune memory and antitumor immunity through dual TRAF2/5–NF-κB and TRAF2–PI3K–AKT signaling pathways. Persistent expression of Survivin together with periodic activation of PKB constitutes a key mechanism supporting the continued functionality of effector T cells, whereas TRAF3 acts as a negative regulator at the TRAF2–NIK signaling node.

#### 3.2.2 OX40 exerts dual roles in Th1/Th2 differentiation and functional maintenance

Th1 cells mediate immunity against intracellular pathogens primarily through the secretion of IFN-γ, TNF-α, and IL-2 (95). In contrast, Th2 cells secrete IL-4, IL-5, and IL-13, which defend against extracellular pathogens and parasites, while also contributing to allergic and atopic diseases (96). OX40 deficiency reduces both Th1- and Th2-associated cytokine levels, underscoring its critical role in helper T cell maintenance (15). However, the precise influence of OX40 on Th1/Th2 differentiation remains debated. In naïve CD4^+^ T cells, OX40 signaling preferentially induces IL-4 production and Th2 polarization; yet, antigen stimulation or IL-12 exposure can override this bias, favoring Th1 differentiation (97). Experimental conditions further shape OX40’s effects: in the absence of adjuvants, OX40 activation promotes Th2 responses, whereas in the presence of adjuvants, it enhances both Th1 and Th2 responses (16). The OX40-OX40L interaction is indispensable for Th2 polarization, as OX40L expressing DCs efficiently drive naïve T cells toward a Th2 phenotype (98). Moreover, OX40 deficiency impairs Th2 clonal expansion and diminishes memory T cell formation and maintenance, reinforcing its pivotal role in Th2 development and function (99, 100).

In the TME, the regulation of Th1/Th2 differentiation by OX40 exhibits pronounced context dependency. Early studies in antigen- and parasite-driven models demonstrated that the OX40-OX40L axis promotes Th2 responses while also supporting Th1 activation, underscoring its essential role in Th2 development (101). This paradigm is further reinforced in TRAF5-deficient models, in which OX40 stimulation markedly increases production of Th2 cytokines such as IL-4 and IL-5, and *in vivo* immunization induces an exaggerated Th2 skewing, indicating that TRAF5 serves as a critical brake on OX40 mediated Th2 programming (102). However, this canonical Th2-favoring pattern undergoes dynamic re-wiring in cancer and chronic inflammatory settings. In hepatitis C virus–associated cirrhosis and tumor tissues, OX40^+^ Tregs are markedly enriched and display a T-bet ^high^ IFN-γ^low^ phenotype associated with suppression of Th1 immunity. In contrast, in non-cirrhotic liver, where IL-12 and IFN-γ concentrations are higher, OX40 signals are counteracted, leading Tregs to convert into Th1-like cells, highlighting the plastic and microenvironment-dependent role of OX40 signaling in modulating the Treg–Th1 axis (103, 104). This immune context dependence is also evident in cutaneous T-cell lymphoma, where OX40L^+^ DCs foster inflammatory T-cell infiltration, and therapeutic intervention can drive a shift from Th2 to Th1 dominated inflammation, illustrating a bidirectional role of OX40 signaling in tumor control and microenvironmental adaptation (105). Consistently, in peripheral T-cell lymphoma, cases characterized by expression of CXCR3, CCR5, or ST2(L) together with OX40 demonstrate better prognosis, further linking OX40 signaling to Th1/Th2 polarization states and tumor-immune dynamics (106).

Overall, OX40 does not operate as a simple binary “switch” directing Th1 or Th2 polarization; rather, it functions as a central immunoregulatory hub shaped by the local cytokine milieu, Treg status, and TRAF-dependent signaling. In non-tumor inflammatory settings, OX40 can support Th2 polarization while concurrently aiding Th1 responses; however, under conditions enriched in IL-12 and IFN-γ, OX40 signaling facilitates the loss of Treg suppressive capacity and drives their conversion toward a Th1-like phenotype. In contrast, within the TME, OX40 signaling is more commonly associated with the accumulation of OX40^+^ Tregs and suppression of Th1 immunity, thereby potentially fostering immune evasion. This bidirectional functional profile underscores the necessity of rational combinatorial strategies when targeting OX40 in cancer immunotherapy, for instance, integrating pro-inflammatory cues such as IL-12, Treg blockade approaches, or modulation of TRAF-dependent pathways to prevent tumor-mediated subversion of OX40 signaling and fully unlock its capacity to potentiate Th1-driven antitumor immunity.

#### 3.2.3 OX40 promotes Th9 cell differentiation via the TRAF6–noncanonical NF-κB pathway

Th9 cells, a recently identified subset of CD4^+^ T cells characterized by IL-9 production, exhibit both antitumor and pro-inflammatory functions (107). Their differentiation is driven by IL-4 derived from Th2 cells in combination with OX40 signaling. OX40 co-stimulation selectively induces robust IL-9 expression in Th9 cells without enhancing Th2- or Th17-associated cytokines (17). Mechanistically, OX40 upregulates TRAF6 and promotes IL-9 production through the noncanonical NF-κB pathway (108). Importantly, OX40 continues to facilitate Th9 differentiation even when the canonical NF-κB pathway is inhibited (17). Under TGF-β–mediated polarization, OX40 ligation suppresses iTreg and Th17 generation, redirecting CD4^+^Foxp3⁻ T cells toward a Th9 phenotype (17). At the level of TME priming, Dectin-1 activated DCs function as potent initiators of Th9 differentiation and drivers of antitumor immunity. Engagement of Dectin-1 upregulates TNFSF15 (TL1A) and OX40L expression on DCs, thereby promoting Th9 polarization through the Syk, Raf1 and NF-κB signaling cascade. In tumor-bearing mice, immunization with such Dectin-1 activated DCs elicits a robust antitumor response that is critically dependent on Th9 cells and IL-9 production (109).

#### 3.2.4 OX40 in the functional regulation of Th17 cells

Th17 cells contribute to host defense and tissue repair through IL-17A secretion but also drive autoimmune pathology (110). OX40 modulates Th17 cell survival and function: in murine autoimmune arthritis models, OX40 enhances IL-17 production (111). In contrast, human studies report that OX40 suppresses IL-17 by upregulating IFN-γand IL-4, while OX40L strongly inhibits IL-17 secretion even in the presence of IL-23, a key inducer of Th17 differentiation (112). These discrepancies indicate that OX40’s regulation of Th17 cells is species- and microenvironment-dependent, necessitating further mechanistic investigation. In the TME, OX40 regulation of Th17 cells shifts from a pro-inflammatory phenotype to an immunosuppressive one. In a DLBCL patient cohort, elevated serum miR-130b was strongly associated with increased IL-17 levels and poor prognosis. Mechanistically, miR-130b targets the IFNAR1/p-STAT1 pathway to downregulate OX40L expression in tumor cells, thereby weakening OX40–OX40L costimulation and maintaining the immunosuppressive phenotype of Th17 cells (113).

#### 3.2.5 OX40 critically regulates the development and function of T follicular helper cells

T follicular helper (Tfh) cells are a specialized subset of CD4^+^ T cells that support B cell differentiation into plasma cells and memory B cells by expressing molecules such as B-cell lymphoma 6 (Bcl6), C-X-C chemokine receptor type 5 (CXCR5), and programmed cell death protein 1 (PD-1) (114). Dysregulated Tfh activity is strongly associated with diseases including lymphoma (115, 116). OX40 plays a pivotal role in Tfh cell development and function. First, OX40 stimulation markedly increases CXCR5 expression, a defining Tfh surface marker, thereby promoting Tfh differentiation (117). Second, OX40 enhances the production of interleukin-21 (IL-21) and CD40L in both naïve and memory Tfh cells, molecules that are essential for Tfh maturation and B cell help (118). Third, OX40 signaling suppresses PR domain zinc finger protein 1 (PRDM1), a transcription factor that inhibits Tfh differentiation, thereby relieving its repression and facilitating Tfh development and function (119). Moreover, aberrant OX40-mediated Tfh responses in systemic lupus erythematosus (SLE) patients contribute to excessive autoantibody production and accelerate disease progression (118). Collectively, OX40’s multifaceted role in Tfh regulation highlights its potential as a therapeutic target and provides mechanistic insights into Tfh-associated diseases.

#### 3.2.6 OX40 signaling in Treg stability and immune remodeling

Tregs are a specialized subset of CD4^+^ T lymphocytes defined by the lineage-determining transcription factor FOXP3, which is essential for maintaining peripheral immune tolerance and preventing excessive inflammation (120–122). Tregs arise through two major developmental pathways: thymus-derived Tregs (tTregs), generated during thymic selection, and peripherally induced Tregs (pTregs/iTregs), which differentiate from conventional CD4^+^ T cells under the influence of environmental cues such as TGF-β and IL-2/STAT5 signaling (123). Functionally, Tregs are indispensable for self-tolerance and immune homeostasis, playing critical roles in preventing autoimmunity, limiting tissue damage during infection, and regulating immune responses in transplantation. However, in the TME, Treg accumulation and enhanced suppressive activity can dampen antitumor immunity, contribute to immune evasion, and correlate with poor clinical outcomes, making Tregs an important target for cancer immunotherapy (124–126).

**Figure 3:**
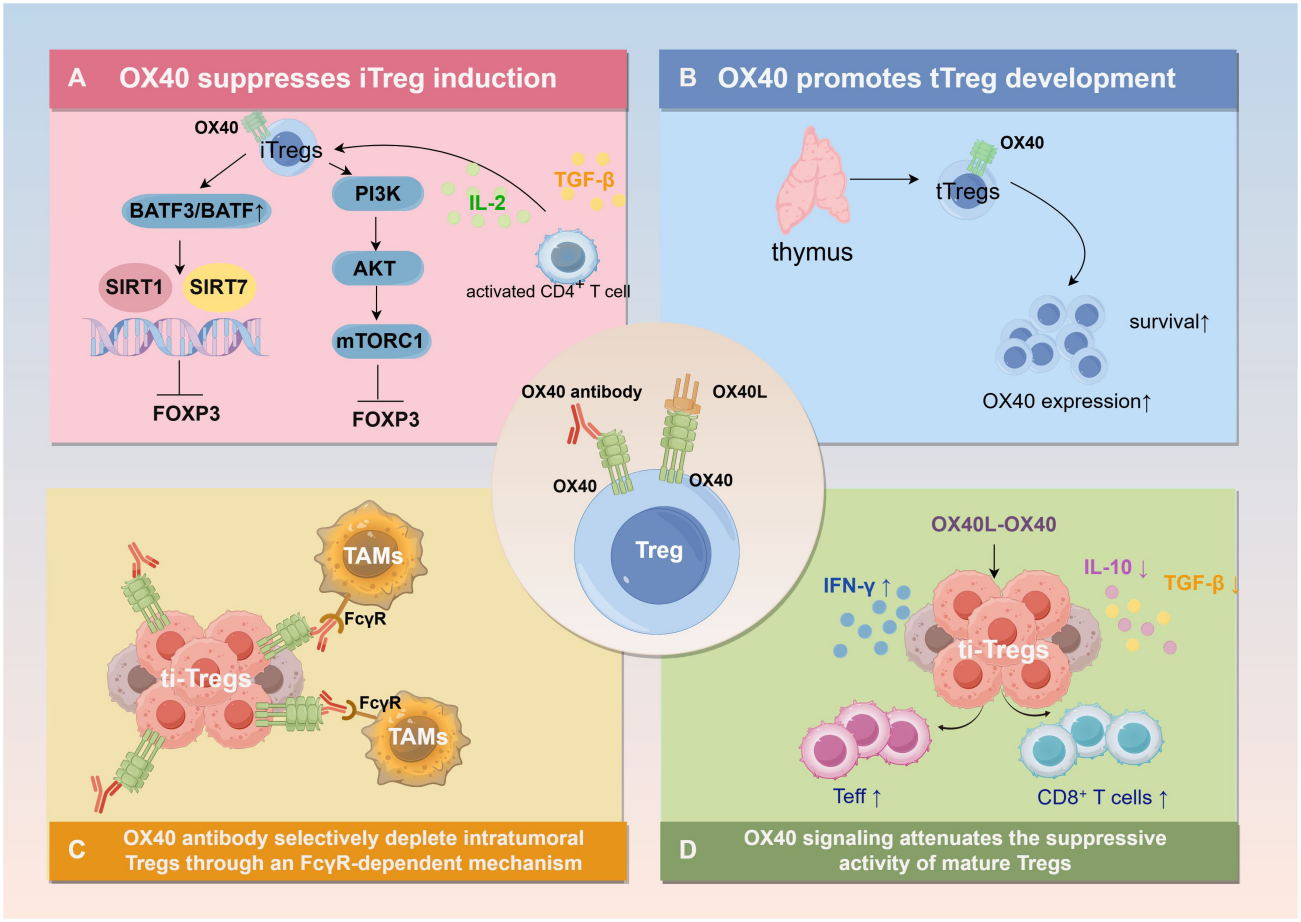
The Effects of OX40 on Tregs. **(A)** OX40 suppresses the proliferation of peripherally induced Tregs (iTregs) by directly modulating FOXP3 expression through the PI3K/AKT/mTORC1 and BATF/SIRT pathways. **(B)** OX40 promotes the expansion of thymus-derived Tregs (tTregs). OX40-deficient mice exhibit reductions in both tTreg precursors and mature cells, whereas OX40L can facilitate tTreg expansion. **(C)** OX40 agonists can selectively deplete intratumoral Tregs through an FcγR-dependent mechanism while simultaneously enhancing CD8^+^ T-cell responses. **(D)** Within the context of tumor immunity, OX40 signaling attenuates the suppressive activity of mature Tregs by reducing IL-10 production, thereby weakening their inhibitory function and ultimately relieving suppression of peripheral effector T cells (Teff). Created with Figdraw.

Within the peripheral immune compartment, Tregs including tTregs and peripherally induced iTregs, express high levels of OX40, positioning them as key targets of OX40-mediated costimulation. Evidence indicates that OX40 signaling within Tregs is not merely an activating cue, but instead exerts bidirectional regulatory functions. On one hand, OX40 supports the survival and functional maintenance of tTregs; indeed, Treg-specific ablation of OX40 disrupts peripheral tolerance and precipitates spontaneous autoimmunity in certain models (127)(**Figure 3B**). On the other hand, OX40 restrains the induction of iTregs, thereby limiting the acquisition of a fully suppressive phenotype by newly generated Tregs in peripheral tissues. Mechanistically, OX40 costimulation inhibits Foxp3 expression and iTreg differentiation by inducing chromatin condensation via BATF3/SIRT1/7 and promoting FoxO1 nuclear exclusion through the AKT/mTOR pathway (128) (**Figure 3A**). Moreover, OX40 signaling in the context of tumor immunity can attenuate the suppressive activity of mature Tregs, reducing IL-10 production and thereby weakening their inhibitory capacity, ultimately relieving suppression of peripheral effector T cells (Teff) (129)(**Figure 3D**).

Importantly, peripheral lymphoid Tregs and tissue-resident or tumor-infiltrating Tregs (ti-Tregs) represent biologically distinct populations with unique transcriptional, metabolic, and functional programs. Recent multi-omics studies have identified conserved ti-Treg signatures across tumor types, highlighting their heightened activation state, tissue-adaptation, and dependence on environmental cues within the TME (130, 131). These differences imply that OX40 signaling may regulate peripheral Tregs and ti-Tregs in fundamentally different ways (132). However, most mechanistic studies evaluating OX40 signaling in Tregs have relied on *in vitro*–stimulated Tregs or splenic Tregs rather than bona fide tumor-infiltrating Tregs. Thus, caution is required when extrapolating findings from peripheral or *in vitro* systems to tissue-resident Tregs within tumors.

Within TME, ti-Tregs commonly exhibit high dependency on and elevated expression of OX40. Studies have demonstrated that CD4^+^FoxP3^+^ Tregs within tumors are predominantly OX40^high^, with markedly higher OX40 expression than their splenic counterparts, indicating a co-stimulatory remodeling program in ti-Tregs that renders them preferential cellular targets for OX40-directed immunomodulation (18). However, it should be noted that earlier *in vivo* studies demonstrated that administration of the OX40 agonist OX86 can lead to expansion of splenic Tregs, indicating that OX40 stimulation does not universally result in intratumoral Treg depletion or suppression (133, 134). Importantly, this peripheral Treg expansion is also dependent on Fcγ receptor engagement, specifically requiring FcγRIIb-mediated antibody cross-linking and receptor clustering to sustain OX40 signaling, rather than representing an FcγR-independent effect. Beyond augmenting effector T-cell activation and memory formation, OX40 signaling plays a critical role in overcoming tumor-induced immune suppression by attenuating Treg function and reducing immunosuppressive pressure within the TME, thereby positioning OX40 as a key target for reversing tumor immune tolerance. Indeed, the OX40 agonist OX86 functionally disables intratumoral Tregs and promotes their depletion, unleashing potent antitumor CD8^+^ T-cell responses. This effect is strictly CD8-dependent, as depletion of CD8^+^ T cells abrogates the therapeutic benefit, underscoring that relief of Treg-mediated suppression enables effector T cells to become the principal mediators of tumor clearance (18). Furthermore, OX86 preferentially eliminates FoxP3^+^ Tregs within tumors through a Fcγ receptor–dependent mechanism, reinforcing that both intratumoral Treg depletion and peripheral Treg expansion rely on FcγR-mediated antibody cross-linking, albeit with distinct contextual and cellular outcomes (135) (**Figure 3C**).

OX40 signaling also remodels the tumor immune microenvironment by promoting the migration of DCs to draining lymph nodes and driving the expansion of tumor-specific cytotoxic T lymphocytes, while simultaneously increasing CD40L expression on effector memory T cells, thereby further amplifying antitumor immune responses (129). Mechanistically, its anti-Treg effects involve markedly suppressing IRF1 and IL-10 expression within tumor-infiltrating Tregs, dismantling their immunosuppressive phenotype and enhancing CD8^+^ T-cell effector function (129). When combined with the chemotherapeutic agent cyclophosphamide (CTX), OX40 stimulation synergistically induces apoptosis of intratumoral Tregs and markedly augments effector T-cell responses, establishing a chemo-immunotherapeutic strategy that potentiates antitumor immunity (136).

In the context of sustaining immune protection and optimizing therapeutic strategies, Fc–OX40L fusion proteins exhibit superior antitumor activity compared with OX86, inducing durable tumor regression and conferring significant survival benefits across multiple murine tumor models (137). Moreover, bifunctional OX40-agonistic fusion proteins, such as OX86–IFN, simultaneously engage OX40 and type I interferon pathways, thereby further potentiating T-cell activation and demonstrating enhanced antitumor and antiviral efficacy relative to monotherapies, underscoring the substantial therapeutic promise of OX40-based immune-enhancing strategies (138).

Taken together, OX40 signaling exerts context-dependent effects on Treg stability and function—supporting thymic tTreg development while limiting peripheral iTreg differentiation—and may additionally promote Fc-dependent Treg clearance in tumors. Optimizing OX40-targeting therapeutics requires consideration of antibody isotype, FcγR engagement, and tissue-specific Treg programs.

### 3.3 The role of OX40 in CD8^+^ T cells

OX40 signaling exerts diverse effects on CD8^+^ T cell-mediated immune responses. Engagement of OX40 agonists remodels the TME by increasing intratumoral antigen-specific CD8^+^ T cells, reducing immunosuppressive populations such as Tregs and myeloid-derived suppressor cells (MDSCs), and downregulating TGF-β expression. Together, these effects enhance CD8^+^ T cell infiltration and cytotoxic activity within tumors (14). Although OX40 deficiency does not affect the initial activation of CD8^+^ T cells, it markedly impairs the generation of both lymphoid and non-lymphoid tissue–resident memory CD8^+^ T cells (89, 139). Evidence from patient-derived tumor-infiltrating lymphocytes (TILs) further demonstrates that OX40 activation augments CD8^+^ T cell antitumor activity and promotes tumor-specific memory responses (140).

Within the TME, OX40 functions as a pivotal costimulatory receptor that enhances CD8^+^ T cell–mediated antitumor immunity through multiple mechanisms. The OX40–TRAF6 axis promotes CTLA-4 ubiquitination and degradation, thereby relieving inhibitory signaling and augmenting CD8^+^ T cell responses (141). In parallel, OX40 signaling sustains CD8^+^ T cell survival and persistence in a Bcl-xL dependent manner (142). In preclinical tumor models, OX40 agonists markedly increase intratumoral CD8^+^ T cell infiltration and concomitantly reduce immunosuppressive components such as MDSCs, TGF-β, and Tregs, resulting in a remodeled immune microenvironment (14). Moreover, combinatorial strategies incorporating OX40 with engineered IL-2 molecules, PD-L1 blockade, or PI3Kβ inhibition exhibit enhanced antitumor synergy, underscoring OX40 as a central immunotherapeutic node and a promising target for combination cancer immunotherapy (140, 143).

## 4 The association between the OX40–OX40L axis and tumor types

OX40 is expressed on TILs across multiple malignancies, including ovarian, head and neck, non-small cell lung cancer (NSCLC), breast, colorectal, hepatocellular, and gastric cancers (144). Given its critical role in modulating antitumor immunity and inflammatory responses, the OX40-OX40L axis has emerged as a promising immunotherapeutic target (145). Considering the heterogeneity of tumor immune microenvironments, OX40 expression and therapeutic responses vary across cancer types. Therefore, we summarize current evidence by tumor entity to highlight disease-specific features and clinical implications. **Table 2** provides an overview of how the OX40–OX40L axis is associated with major tumor types across current clinical and translational studies.

**Table 2:** Summary of the association between the OX40-OX40L axis and major tumor types.

Tumor type	OX40/OX40L expression pattern	Immune microenvironment features	Clinical/prognostic evidence	Key implications
NSCLC, SCLC	OX40 widely expressed (~90% NSCLC); OX40L low (~10%); subtype-dependent	Enriched in CD4^+^/CD8^+^ T cells and cDC1s; expression at tumor–stromal interface	Prognosis inconsistent across cohorts; high OX40 predicts improved survival in SCLC	High OX40 suggests inflamed TME; rational target for combination immunotherapy
TNBC	OX40/OX40L expressed on TILs; OX40 enriched on Tregs	Co-occurs with immune activation transcripts; balance of Treg vs. effector OX40^+^ subsets critical	Prognostic effect dependent on cellular composition; Treg-dominated OX40 may indicate poor outcome	Cellular-resolution OX40 profiling required; strong rationale for OX40 agonism + PD-1/RT
HNSCC	Robust OX40 expression on CD4^+^ and CD8^+^ TILs	Promotes expansion of tumor-reactive CD103^+^CD39^+^CD8^+^ T cells; OX40^+^ pDC–cDC cooperation	Neoadjuvant OX40 agonism expands tumor-reactive clones; responders show enhanced TRM-like CD8^+^ cells	OX40 enhances tumor-specific immunity; may predict response to immunotherapy
CRC	Strong OX40 gene and protein expression; correlated with CD8 and FOXP3	Co-enrichment with CD16^+^ myeloid cells; strong effector + regulatory T-cell signature	High OX40 and CD8 → survival comparable to stage I; circulating OX40^+^ Tregs predictive (AUC≈0.92)	High value as prognostic marker; strong synergistic potential with PD-1/PD-L1 blockade
GC	High OX40 in early-stage (I–II) tumors; reduced in late-stage tumors	Reflects immune-competent microenvironment early in disease	High OX40 on TILs predicts improved survival with nivolumab	OX40 as biomarker for immunotherapy benefit and early immune competence
HCC	OX40 mRNA elevated in tumors; OX40L low; associated with AFP and vascular invasion	Immune-tolerant TME; OX40 involved in iNKT cell pyroptosis	High OX40 linked to poor differentiation and decreased OS	Requires careful therapeutic modulation; OX40L mRNA vaccines show preclinical benefit
PDAC	OX40^+^ cells ≈10% of infiltrating immune cells	Present in spatially defined immune niches	High OX40 predicts favorable prognosis, especially in PD-L1–negative tumors	Potential biomarker for stratification; early-stage therapeutic relevance

### 4.1 Thoracic tumors (NSCLC and SCLC)

OX40 is expressed in ~90% of NSCLC cases, with subtype specificity, whereas OX40L expression is observed in only ~10%, indicating receptor-ready yet ligand-limited activation (146). OX40 signaling is enriched at tumor-mesenchymal junctions, consistent with localized immune infiltration (146). OX40 expression correlates with infiltration of CD4^+^αβ T cells, CD8^+^αβ T cells (77), and conventional type 1 dendritic cells (cDC1s) (147), suggesting an association with inflamed TME and enhanced antitumor immunity. Prognostic associations remain heterogeneous. In a cohort of 139 NSCLC patients, lower OX40 expression in TILs was associated with prolonged OS and RFS (148), whereas in 100 stage I–III NSCLC patients, high OX40 expression predicted improved survival (145). These discrepancies may reflect differences in immune-cell subset stratification or patient cohort heterogeneity. Notably, OX40/OX40L expression inversely correlates with PD-1/PD-L1 expression (148), indicating a mechanistically distinct pathway that may provide therapeutic benefit independent of PD-1 blockade.

In SCLC, high OX40 expression is associated with improved clinical outcomes: median RFS was 26.0 months vs. 13.2 months in low-expression cases, and median OS was not reached in the high-expression group vs. 29.8 months in the low group. Multivariate analysis confirmed OX40 as an independent predictor of prolonged RFS, suggesting a role in suppressing early recurrence and informing immunotherapy development (149).

Collectively, thoracic tumors demonstrate high OX40 expression, enrichment of effector immune cell infiltration, and favorable prognostic implications in select contexts. These findings support OX40 as both a biomarker and a rational target for combination immunotherapy strategies in lung cancer.

### 4.2 Breast tumor

OX40 and OX40L protein expression has been detected on TILs in human breast cancer, including triple-negative breast cancer (TNBC). In certain patient cohorts, OX40 expression is associated with immune-activation signatures, and elevated OX40 levels may correlate with malignant transformation, tumor progression, invasiveness, and metastasis in breast cancer biology (150). At the immunophenotypic level, OX40 expression is relatively enriched on Tregs, suggesting that high OX40 expression dominated by Tregs may be linked to unfavorable clinical outcomes; therefore, delineating the cellular composition of OX40-positive subsets is critical for accurately interpreting their clinical significance (77, 151). In inflammatory immune microenvironments characteristic of breast cancer, particularly TNBC, OX40/OX40L expression frequently co-occurs with immune activation-related transcripts (152). However, the prognostic effect of OX40 depends on the lineage distribution of OX40^+^ cells and the contextual immune milieu, underscoring the importance of distinguishing Treg-dominated versus effector T cell-dominated OX40 expression profiles (86, 153).

In preclinical murine breast tumor models, treatment with agonistic monoclonal antibodies targeting OX40 or 4-1BB significantly suppressed primary tumor growth and reduced metastatic burden (154). Additional studies demonstrated that OX40 activation mediated by M1-like macrophage-derived extracellular vesicles enriched in OX40L not only augmented adaptive immune responses but also reprogrammed M2-like tumor-associated macrophages toward an M1 phenotype, collectively resulting in robust inhibition of tumor progression and metastasis through activation of the OX40/OX40L signaling axis (155). Moreover, in murine TNBC models, combined administration of agonistic anti-OX40 stimulation with PD-1 blockade and radiotherapy markedly increased the intratumoral ratio of CD8^+^ T cells to CD4^+^FOXP3^+^ Tregs, substantially reduced the proportion of exhausted CD8^+^ T cells, and achieved durable tumor control, with long-term survival exceeding 60% beyond two months (156).

Multiple OX40 agonists have been evaluated in phase I/Ib and I/II clinical trials involving patients with advanced solid tumors, including breast cancer. Overall, these agents have demonstrated acceptable safety profiles and clear pharmacodynamic evidence of immune activation, yet objective responses to monotherapy have been limited. Consequently, recent studies have shifted toward rational combination strategies involving PD-1/PD-L1 or CTLA-4 blockade, radiotherapy, or cancer vaccines (157, 158).

### 4.3 Head and neck tumors

In head and neck squamous cell carcinoma (HNSCC), OX40 is expressed on TILs, and its activation is associated with enhanced antitumor immune activity (159). The upregulation of OX40 within both CD4^+^ and CD8^+^ T-cell subsets indicates an active immune response in the TME, providing a strong rationale for considering OX40 as a potential therapeutic target.

Following treatment with OX40 agonists, tumor tissues from patients with HNSCC exhibited a marked clonal expansion of effector T cells, accompanied by increased frequencies of activated CD4^+^ and CD8^+^ TILs [147]. Notably, approximately 25% of patients showed significant enrichment of CD103^+^CD39^+^CD8^+^ T cells after therapy—cells characterized by tissue-resident and cytotoxic features. The accumulation of this subset correlated with recurrence-free status and has been proposed as a potential biomarker of therapeutic response (160). Mechanistically, OX40^high plasmacytoid dendritic cells (pDCs) cooperate with conventional cDCs to activate tumor antigen–specific CD8^+^ T cells and mediate direct cytotoxicity (147). Moreover, in squamous cell carcinoma models, OX40 agonists have been shown to reverse Treg-mediated immunosuppression and promote the proliferation of tumor-reactive CD4^+^ T cells (79).

At the preclinical level, studies in squamous carcinoma-related models have demonstrated that OX40 agonism can reverse Treg-mediated immunosuppression and enhance tumor-reactive T-cell responses, providing a strong rationale for its clinical application either as monotherapy or in combination with PD-1 blockade, radiotherapy, or cancer vaccines (79). In a phase Ib neoadjuvant trial using the anti-OX40 antibody MEDI6469, patients with locally advanced HNSCC received MEDI6469 prior to curative surgery, showing favorable safety and feasibility. Treatment induced increased activation and clonal expansion of peripheral and intratumoral CD4^+^ and CD8^+^ T cells. Notably, approximately 25% of patients exhibited postoperative enrichment of CD103^+^CD39^+^ tumor-reactive CD8^+^ TILs, all of whom remained recurrence-free during follow-up, suggesting that OX40 agonism can potentiate antitumor immunity in HNSCC (160). Within the HNSCC TME, OX40^high^ pDCs cooperate with conventional cDCs to promote antigen-specific CD8^+^ T-cell responses and cytotoxicity, in animal models, elevated OX40^+^ pDC levels are required to restrain tumor growth, highlighting the OX40^+^ pDC–cDC network as a critical node mediating the OX40 axis antitumor effects in HNSCC (147).

Studies in HNSCC and cutaneous squamous cell carcinoma have demonstrated that OX40 signaling enhances effector T-cell activity, expands tumor-specific T-cell clones, promotes the formation of tissue-resident–like CD8^+^ T-cell populations, and modulates dendritic cell cooperation, thereby remodeling the tumor immune microenvironment and eliciting robust antitumor effects.

### 4.4 Gastrointestinal tumors (colorectal and gastric cancer)

The expression of OX40 in colorectal cancer (CRC) is closely associated with CD8^+^ lymphocyte infiltration and favorable clinical outcomes. Large-scale cohort analyses of colon cancer specimens have demonstrated a strong correlation between OX40 gene expression and the expression of CD8 and FOXP3, indicating its involvement in both effector and regulatory T-cell compartments. Patients with high OX40 expression exhibited significantly better prognosis in univariate analyses, and those with concomitantly high OX40 and CD8 infiltration (OX40^high^/CD8^high^) showed overall survival comparable to that of stage I CRC, identifying OX40 as an independent favorable prognostic biomarker (161). Moreover, concomitant infiltration of OX40^+^ and CD16^+^ immune cells further reinforced survival benefit, as high densities of these two subsets were independently associated with improved outcomes; notably, CD16^+^ myeloid and innate immune cells alone also correlated with longer survival (162). In addition, circulating CD30^+^OX40^+^ Tregs demonstrated excellent diagnostic and prognostic performance (ROC AUC ≈ 0.92), suggesting that OX40 serves not only as a marker of intratumoral immune activation but also as a potential biomarker for systemic immune monitoring (153).

In preclinical models of CRC, OX40 agonism has demonstrated pronounced synergistic antitumor effects when combined with PD-1/PD-L1 blockade. In a humanized OX40 knock-in mouse model bearing MC38 colon tumors, administration of an agonistic anti-OX40 antibody not only enhanced antigen-specific CD8^+^ T-cell cytotoxicity and IFN-γ production but also effectively reduced the intratumoral proportion of Tregs, thereby significantly improving the therapeutic response to anti-PD-1 treatment (163). This finding indicates that OX40 co-stimulation and immune checkpoint blockade act through complementary mechanisms: their combination amplifies effector T-cell activation while concurrently relieving immunosuppression. Further evidence from the CT26 colon carcinoma model introduced a molecularly integrated strategy, in which a PD1-Fc-OX40L fusion protein simultaneously mediates PD-1 inhibition and OX40 activation within a single construct, resulting in marked tumor growth suppression and prolonged survival (164).

Multiple studies have demonstrated that OX40 expression is closely associated with immune response activity and clinical outcomes. In patients with advanced gastric cancer (GC) treated with the anti–PD-1 antibody nivolumab, high OX40 expression on tumor-infiltrating T cells was significantly correlated with improved survival, suggesting that OX40 may serve as a potential biomarker predictive of immunotherapy benefit (165). In addition, clinical investigations have revealed elevated OX40 expression within the gastric mucosal immune microenvironment of GC patients, with markedly higher levels observed in stage I–II tumors compared with stage III–IV disease. This stage-dependent decline indicates that immune function becomes progressively suppressed during disease progression and implies that OX40 plays an important immunomodulatory role within the gastric tumor micro-environment (166).

Overall, OX40 functions as a central immunoregulatory node across gastrointestinal cancers, linking effective T-cell activation with the reversal of immune suppression. Its consistent association with improved prognosis and responsiveness to immunotherapy in both colorectal and gastric cancer underscores its translational potential as a therapeutic target. Future strategies should focus on context-specific OX40 agonism—particularly in combination with PD-1/PD-L1 blockade—to optimize antitumor immunity and overcome resistance within the gastrointestinal TME.

### 4.5 Hepatocellular carcinoma

Multiple cohort studies have demonstrated that OX40 mRNA expression in tumor tissues is significantly higher than that in adjacent non-tumor or normal hepatic tissues, whereas OX40L mRNA levels are comparatively lower. Elevated OX40 expression was markedly associated with the degree of tumor differentiation, and showed a non-significant trend toward shorter recurrence-free survival (167). These findings suggest that dysregulated expression of OX40 and OX40L within the hepatocellular carcinoma (HCC) microenvironment may induce immunosuppression and consequently promote tumor progression. Furthermore, high TNFRSF4 expression has been linked to distinct immune infiltration patterns and mutational landscapes, indicating its potential as a combined biomarker for prognosis and immune subtyping (168). Elevated OX40 expression in HCC has also been correlated with high serum alpha-fetoprotein (AFP) levels, vascular invasion, and shorter overall survival (169).

Preclinical studies have shown that hepatic invariant natural killer T (iNKT) cells express the costimulatory TNF superfamily receptor OX40, and that OX40 stimulation triggers severe pyroptosis of iNKT cells, accompanied by the release of pro-inflammatory cytokines leading to liver injury (170). In addition, an innovative therapeutic approach using an mRNA vaccine encoding OX40L delivered via lipid nanoparticles (LNPs) significantly suppressed tumor growth and prolonged survival in an H22 murine HCC model, along with marked increases in CD4^+^ and CD8^+^ T-cell infiltration (171). These results highlight the therapeutic potential of ligand-targeted OX40 activation strategies in HCC, although further HCC-specific evidence remains to be established.

In future studies, patient stratification based on OX40/OX40L expression and the Treg–effector T-cell balance should guide the development of precision predictive models for therapy response. Combination regimens integrating OX40 agonists with PD-1 inhibitors, 4-1BB, or TLR agonists may enhance antitumor immunity and overcome monotherapy limitations. Considering the immune-tolerant liver environment, careful dose and schedule optimization is required to minimize hepatotoxicity while maintaining efficacy.

### 4.6 pancreatic ductal adenocarcinoma

In pancreatic ductal adenocarcinoma (PDAC), research on the expression of OX40 remains less extensive compared with that on other immune checkpoints; however, available evidence has begun to elucidate its spatial distribution and potential functional significance within the tumor immune microenvironment. Studies have shown that OX40^+^ immune cells constitute approximately 10.2% of tumor-infiltrating immune cells in PDAC. Moreover, high OX40 expression has been identified as an independent favorable prognostic factor, with particularly pronounced prognostic value in patients lacking PD-L1 expression (172).

## 5 Current status of clinical studies targeting OX40 in cancer therapy

### 5.1 Monotherapy

Early clinical trials of OX40 agonists have defined their safety profile, pharmacodynamic characteristics, and the practical limits of monotherapy efficacy. Across multiple solid tumor types, these agents demonstrate consistent tolerability and robust immune activation, yet their objective response rates remain modest, indicating that OX40 stimulation is better positioned as a foundational component of combination immunotherapy rather than a stand-alone therapeutic strategy.

The first-in-human study of the murine IgG1 antibody MEDI6469 (NCT01644968) established the feasibility of OX40 agonism in patients with melanoma, colorectal cancer, and renal cell carcinoma. Treatment produced no dose-limiting toxicities, enhanced antigen-specific T- and B-cell responses, and upregulated OX40 expression on Tregs. Although RECIST responses were infrequent, approximately 40% of patients exhibited reductions in metastatic lesions, providing early clinical signals of activity (90). In a neoadjuvant study in head and neck squamous cell carcinoma (NCT02274155), short-course MEDI6469 further increased peripheral and intratumoral CD4^+^/CD8^+^ T-cell proliferation and enriched CD103^+^CD39^+^CD8^+^ TILs, with no recurrence observed during follow-up (160).

Subsequent development of humanized OX40 agonists including MEDI0562, MOXR0916, ivuxolimab (PF-04518600), and INCAGN01949—confirmed these findings in broader cohorts of heavily pretreated patients (158, 173–175). All agents displayed favorable safety without reaching maximum tolerated doses; adverse events were predominantly grade 1–2 fatigue, rash, nausea, and infusion-related reactions. Pharmacokinetic and pharmacodynamic analyses revealed dose-proportional exposure, >80–90% receptor occupancy, peripheral T-cell activation, PD-L1 upregulation, and expansion of CD4^+^ memory T-cell subsets, demonstrating effective engagement of OX40 signaling.

Despite this consistent immune activation, clinical responses remained limited. Across trials, objective response rates ranged from 1–6%, with most patients achieving only stable disease. Occasional lesion shrinkage and durable disease control tended to occur in tumors with pre-existing immune infiltration, highlighting the dependence of OX40 agonism on a permissive immunologic baseline.

INBRX-106 is an engineered hexavalent OX40 agonist antibody developed to overcome the limitations of conventional bivalent OX40 agonists, which inadequately induce receptor clustering and consequently fail to generate optimal downstream signaling or clinical efficacy. By virtue of its hexavalent architecture, INBRX-106 achieves high-order OX40 receptor clustering independent of Fc-mediated crosslinking, leading to markedly enhanced signal transduction. Preclinical studies demonstrate that INBRX-106 induces stronger NF-κB activation, T-cell proliferation, and effector function compared with bivalent agonists, and produces significant tumor regression and survival benefits across multiple murine tumor models. Findings from the phase I/II clinical trial (NCT04198766) further validate these mechanistic advantages, showing robust T-cell activation and expansion, particularly within central and effector memory compartments (59).

Collectively, these data depict a pattern of strong pharmacodynamic activity but restricted monotherapy efficacy, consistent with the biological role of OX40 as an amplifier of existing T-cell immunity rather than an initiator of *de novo* responses. Consequently, OX40 agonists are now regarded as promising platform agents for combination therapies—particularly with PD-1/PD-L1 or CTLA-4 blockade, CD137 agonists, or IL-2 variants. Future investigations should prioritize optimal dosing schedules, treatment sequencing, biomarker selection, and rational multi-pathway co-activation strategies to fully exploit the immunopotentiating potential of OX40 signaling. A comprehensive overview of these early-phase OX40 agonist trials including study design, patient characteristics, dosing strategies, safety findings, and efficacy outcomes is summarized in **Table 3**.

**Table 3:** Clinical studies of OX40-targeted monotherapy in cancer therapy.

NCT number	Drug and isotype type	Study design	Population and dosing regimen	Primary endpoint and key findings	Safety and efficacy signals	Refrence
NCT01644968	MEDI6469 (murine IgG1 agonist mAb)	Phase I, monotherapy, dose-escalation study	30 patients with advanced solid tumors (melanoma, colorectal cancer, renal cell carcinoma); 0.1–10 mg/kg IV, every 3 weeks	Enhanced T- and B-cell antigen responses; upregulation of OX40 on CD4^+^FoxP3^+^ Tregs	No dose-limiting toxicity; 40% showed reduction of metastatic lesions	(90)
NCT02274155	MEDI6469 (murine IgG1 agonist mAb)	Phase Ib, neoadjuvant study	16 patients with locally advanced HNSCC; administered for 2 weeks prior to surgery	Increased peripheral CD4^+^/CD8^+^ T-cell proliferation; activated CD4^+^ TILs increased in 80% of patients	25% (4/16) had enrichment of CD103^+^CD39^+^CD8^+^ TILs; all remained recurrence-free during 24-month median follow-up	(160)
NCT02318394	MEDI0562 (humanized IgG1 agonist mAb)	Phase I, monotherapy, dose-escalation study	55 patients with advanced solid tumors; 0.1–3 mg/kg IV, every 3 weeks	Evidence of immune activation with partial responses observed (2 PRs)	67% experienced manageable TRAEs (fatigue, infusion reactions); disease control rate (DCR) 44%	(158)
NCT04730843	ES102 (humanized IgG1 agonist mAb)	Phase 1, Dose-escalation study	18 patients with advanced solid tumors;	Primary endpoint: safety, DLT, MTD/RP2D; secondary endpoints: ORR, DCR, PK/PD, and immune biomarkers	No Study Results Posted	
NCT02219724	MOXR0916 (humanized anti-OX40 IgG1 mAb)	Phase I, open-label, dose-escalation study	Patients with advanced or metastatic solid tumors; IV infusion every 3 weeks	Evaluated safety, pharmacokinetics, and preliminary efficacy	Well tolerated; no DLT observed; clinical responses rare, mainly disease stabilization	(173)
NCT02923349	INCAGN01949 (fully human IgG1 agonist mAb)	Phase I/II, open-label, dose-escalation and expansion	Advanced or metastatic solid tumors (subtypes including endometrial, ovarian, renal, melanoma, NSCLC); IV dosing ≥200 mg	Achieved >90% receptor occupancy; limited peripheral T-cell activation; no consistent increase in intratumoral effector T cells	Acceptable safety; limited single-agent activity	(174)
NCT02315066	PF-04518600 (ivuxolimab) (human IgG2 anti-OX40 mAb)	Phase I, multicenter, open-label, dose-escalation study	Patients with advanced or metastatic solid tumors; 0.01–10 mg/kg IV, every 2 weeks	Primary endpoint: safety and tolerability; secondary endpoints: preliminary antitumor activity and biomarker evaluation	Manageable safety profile; early signals of clinical and pharmacodynamic activity	(175)
NCT04198766	INBRX-106 (hexavalent agonist based on engineered IgG1 scaffold)	Phase I/II, open-label, dose-escalation and expansion study	Patients with advanced/metastatic solid tumors; Multiple dose levels administered intravenously	Safety and tolerability as primary endpoints;	Well tolerated with no unexpected toxicities;	(59)

### 5.2 Combination immunotherapy

Preclinical studies have consistently shown that OX40 agonists, when combined with PD-1 or CTLA-4 blockade, elicit marked immunologic synergy, characterized by enhanced activation of CD4^+^ and CD8^+^ T cells and improved tumor clearance. These findings positioned OX40-based combinations as a promising immunotherapeutic strategy (176, 177). However, once translated into clinical trials, the therapeutic performance has fallen substantially short of expectations. Across multiple phase I/II studies evaluating OX40 agonists in combination with PD-1/PD-L1 inhibitors—most notably GSK3174998, BMS-986178, and MOXR0916—the overall response rates have remained uniformly low.

For example, GSK3174998, administered alone or with pembrolizumab in 138 patients with advanced solid tumors, demonstrated linear pharmacokinetics and >80% target receptor occupancy. Nevertheless, treatment-related adverse events (TRAEs) occurred in 51-64% of patients, predominantly fatigue, and the disease control rate (DCR) was only 9%, offering no improvement over historical outcomes with PD-1 monotherapy (157). Similarly, the phase I/IIa trial of BMS-986178, alone or in combination with nivolumab/ipilimumab (n=165), revealed manageable toxicity with rare grade 3–4 events but failed to produce objective responses (178). The MOXR0916 plus atezolizumab study reported minor early signals of antitumor activity in a subset of patients but showed no dose-limiting toxicities and only limited overall response rates, indicating that the regimen was safe but clinically unimpressive (179). Collectively, these data suggest that, despite adequate receptor engagement and evidence of peripheral immune activation, the expected synergy between OX40 agonism and PD-1/PD-L1 inhibition has not translated into meaningful clinical benefit.

A similar disconnect between immune activation and clinical efficacy is observed with OX40–CTLA-4 combinations. In trials evaluating BMS-986178 with ipilimumab, modest T-cell activation was detected. Likewise, in studies of MEDI0562 combined with tremelimumab, proliferating Ki67^+^CD4^+^ and Ki67^+^CD8^+^ memory T cells increased by more than 100%, accompanied by declines in OX40^+^FOXP3^+^ Tregs in some patients (180). Despite these pronounced peripheral immunologic changes, no meaningful objective responses were observed, and the clinical activity remained far below preclinical expectations. Several mechanisms may underlie this discrepancy, including the failure to achieve an optimal temporal window for simultaneous OX40 activation and CTLA-4 blockade, compensatory immunosuppressive pathways, and limited T-cell reserves in heavily pretreated patients.

The combination of OX40 and 4-1BB agonists—a dual costimulatory strategy that demonstrates potent synergy in murine models—has also yielded disappointing clinical outcomes. In the phase I study of ivuxolimab plus utomilumab (n = 57), the disease control rate was 35.1%, but objective responses occurred in only 3.5% of patients, and the expansion cohort displayed similarly limited partial responses (181). Although the regimen was moderately well tolerated and exhibited dose-dependent increases in drug exposure, it failed to reproduce the robust T-cell expansion and tumor eradication observed in preclinical systems.

In patients with low-grade B-cell lymphoma, the triplet regimen of low-dose radiotherapy, intratumoral TLR9 agonist SD101, and OX40 agonist BMS-986178 also failed to produce the anticipated synergy. In this phase I study, only one patient achieved a partial response and nine maintained stable disease, yielding outcomes inferior to those historically achieved with radiotherapy plus TLR9 agonist alone (182). Flow cytometry and single-cell transcriptomic analyses demonstrated activation of T cells and NK cells following treatment; however, high baseline OX40 expression correlated with inferior progression-free survival (<6 months), suggesting that the clinical performance of OX40 agonists is highly dependent on the patient’s pre-existing immune landscape rather than on target engagement alone.

A separate study evaluating MEDI0562 in combination with durvalumab reported TRAEs in 74.1% of patients, primarily grade 1–2 events. Although the regimen substantially increased memory CD4^+^ and CD8^+^ T-cell proliferation and reduced Treg frequencies in some individuals, objective tumor responses remained exceedingly rare (180). These findings reinforce that strong immune activation does not necessarily translate into meaningful clinical efficacy, and that OX40 agonists are constrained by several biological limitations, including tumor microenvironmental suppression, T-cell exhaustion, and suboptimal Fc-mediated cross-linking.

BGB-A445 is a non–ligand-competitive OX40 agonist antibody that robustly activates T cells while preserving the natural OX40–OX40L interaction and maintaining dendritic cell function. Its IgG1 Fc domain confers ADCC activity, enabling dose-dependent depletion of tumor-infiltrating Tregs and thereby augmenting antitumor immunity (183). In a phase I clinical study (NCT04215978), BGB-A445 was well tolerated with no dose-limiting toxicities, achieved saturated receptor occupancy at doses ≥300 mg, and induced clear on-target immune activation and early antitumor signals, including a confirmed ORR of 21.3% in the combination cohort. Collectively, these findings indicate that BGB-A445, through its unique non-blocking agonistic mechanism and Treg-depleting capacity, offers superior immunostimulatory activity and promising clinical potential compared with conventional OX40 agonists (184).

Overall, early-phase trials show that OX40-based combination immunotherapy is well tolerated but delivers limited clinical benefit, revealing a recurring gap between pharmacodynamic activity and durable tumor responses. Major obstacles include the transient kinetics of OX40 expression, insufficient activatable CD4^+^ T-cell pools in advanced disease, high Treg abundance with compensatory suppression, and suboptimal FcγR-mediated cross-linking of certain antibodies. Future progress will require improved Fc engineering, dosing schedules tailored to OX40 dynamics, biomarker-driven patient selection, and rational combinations with vaccines, metabolic modulators, or locoregional treatments to better harness OX40’s therapeutic potential. The key clinical trials discussed above are systematically summarized in **Table 4**, which compares study design, dosing regimens, safety profiles, and clinical outcomes.

**Table 4:** Clinical studies of OX40-targeted combination immunotherapy in cancer therapy.

NCT number	Drug and type	Combination therapy	Study design	Population and dosing regimen	Primary endpoint and key findings	Safety and efficacy signals	Refrence
NCT04116710	HS-130 (allogeneic cell-based vaccine expressing OX40L–human IgG1 Fc fusion)	Viagenpumatucel-L (HS-110)	Phase I, open-label, dose-escalation	~15 patients with advanced solid tumors; intradermal HS-130 + HS-110 every 2 weeks	No Study Results Posted	No Study Results Posted	
NCT02737475	BMS-986178 (humanized IgG1 agonistic anti-OX40 mAb)	nivolumab and/or ipilimumab	Phase I/IIa, open-label	165 patients with advanced solid tumors;Standard phase I dose-escalation and expansion regimens	Safety was the primary endpoint; treatment was well tolerated but showed no objective responses or meaningful antitumor activity	Common TRAEs (≥5%) included fatigue, pruritus, and rash; grade 3–4 AEs occurred in 5% (monotherapy) and 8–15% (combination), with no discernible efficacy signals.	(178)
NCT03410901	BMS-986178 (humanized IgG1 agonistic anti-OX40 mAb)	SD-101(Intratumoral TLR9 agonist)+ Low-dose radiation	Phase I, single-arm, dose-escalation/safety study evaluating tolerability	14 adults with low-grade B-cell lymphoma	Safety, tolerability, and DLT assessment;No dose-limiting toxicities; safety profile consistent with prior SD-101 + radiation experience;1 partial response, 9 stable disease	Generally well tolerated; no new synergistic toxicities;Weak clinical signal relative to strong preclinical results	(182)
NCT02705482	MEDI0562 (humanized IgG1 agonistic anti-OX40 mAb)	Durvalumab or Tremelimumab	Phase I, multicenter	Advanced solid tumors; IV MEDI0562 q2w (mono or combination)	Demonstrated peripheral T-cell proliferation and immune activation	Well tolerated; low ORR (<5%)	(180)
NCT03092856	PF-04518600 (ivuxolimab; human IgG2 agonistic anti-OX40 mAb)	Axitinib	Phase II, randomized	Metastatic RCC; ivuxolimab + axitinib vs axitinib alone	No Study Results Posted	No Study Results Posted	
NCT02528357	GSK3174998 (humanized IgG4 S228P agonistic anti-OX40 mAb)	Pembrolizumab (optional)	Phase I, first-in-human	Advanced solid tumors; dose-escalation ± pembrolizumab	Demonstrated target engagement and immune activation	Safe; minimal ORR; development discontinued	(157)
NCT02315066	PF-04518600 (ivuxolimab; human IgG2 agonistic anti-OX40 mAb)	Utomilumab (4-1BB agonist)	Phase I, multicenter	Advanced/metastatic solid tumors; monotherapy dose escalation or combination	Identified RP2D; modest immune activation	Well tolerated; no meaningful objective responses	(181)
NCT01862900	MEDI6469 (murine IgG1 agonistic anti-OX40 mAb)	SBRT (radiotherapy)	Phase I/II, open-label	Metastatic breast cancer; IV MEDI6469 + SBRT	No Study Results Posted	No Study Results Posted	
NCT02221960	MEDI6383 (OX40L–IgG4 Fc fusion protein)	Durvalumab (optional)	Phase I, multicenter	Locally advanced/metastatic solid tumors; dose-escalation ± durvalumab	No Study Results Posted	No Study Results Posted	
NCT02410512	MOXR0916 (humanized IgG1 agonistic anti-OX40 mAb)	Atezolizumab: (Anti-PD-L1 humanized IgG1 mAb)	Phase I, open-label, multicenter, 3 + 3 dose-escalation with expansion cohort	Advanced/metastatic solid tumors; MOXR0916 0.8–600 mg q3w + fixed 1200 mg atezolizumab q3w. Selected expansion dose: MOXR0916–300 mg + atezolizumab 1200 mg	No DLTs; PK consistent with single agents; early objective responses observed	Well tolerated; mostly Grade 1 AEs; one Grade 3 pneumonitis; preliminary antitumor activity noted	(179)
NCT04215978	BGB-A445 (non–ligand-competitive OX40 agonist; human IgG1 Fc with ADCC activity)	Tislelizumab (anti–PD-1 antibody)	Phase I, open-label, dose-escalation study evaluating safety, PK/PD, and preliminary antitumor activity	Patients with previously treated advanced solid tumors; IV BGB-A445 across multiple dose levels (≥300 mg achieving receptor saturation), alone or in combination with tislelizumab	saturated OX40 receptor occupancy ≥300 mg; robust on-target immune activation; early antitumor signals including 21.3% confirmed ORR in combination cohort	Well tolerated with no dose-limiting toxicities; clear immune activation and preliminary antitumor activity	(184)

### 5.3 Bispecific antibody

Bispecific antibodies (BsAbs) that simultaneously target OX40 and additional immunoregulatory molecules (such as PD-L1, CD137, or CTLA-4) enable more efficient receptor clustering and integrated signaling within the TME. This design helps overcome several limitations associated with conventional OX40 agonists, including FcγR-dependent crosslinking, insufficient *in vivo* activation, and suppression mediated by Tregs. Across available preclinical and early-phase clinical studies, OX40-based BsAbs generally exhibit stronger immunostimulatory activity and superior antitumor potential compared with OX40 monotherapies.

Among FcγR-independent dual-agonistic BsAbs, the OX40/CD137 bispecific antibody FS120 represents a key example. Whereas traditional TNFRSF agonists rely heavily on FcγR-mediated crosslinking, FS120 promotes CD4^+^ and CD8^+^ T-cell activation through cooperative engagement of its two stimulatory targets in the absence of FcγR involvement. Its murine surrogate has demonstrated robust antitumor efficacy across multiple syngeneic tumor models, alongside markedly reduced hepatic T-cell infiltration relative to CD137 monotherapy—indicating a potentially improved safety profile. This FcγR-independent dual-activation mechanism provides strong rationale for its continued clinical development (185).

Another major class of OX40 BsAbs employs PD-L1-mediated spatial clustering to enhance OX40 activation. By fusing two PD-L1-binding VHH fragments to the C-terminus of a non-blocking OX40 agonistic antibody, investigators generated a tetravalent PD-L1/OX40 BsAb capable of promoting localized OX40 clustering on PD-L1–expressing cells. This molecule retains both PD-L1 blockade and OX40 agonism, while markedly amplifying T-cell activation in the presence of PD-L1 and producing antitumor responses superior to either parental antibody alone or in combination across multiple *in vivo* models. Such PD-L1-dependent crosslinking strategies may enhance tumor-selective activation while mitigating systemic toxicity (186).

Following this design concept, the novel Fc-silent tetravalent PD-L1/OX40 BsAb EMB-09 has demonstrated further strengthened immune activation and *in vivo* activity. EMB-09 concurrently blocks PD-1/PD-L1 interactions and induces PD-L1-dependent OX40 stimulation, yielding antitumor efficacy superior to anti-PD-L1 monotherapy. It enhances effector-memory T-cell activity, promotes infiltration of stem-like CD8^+^ T cells, and drives a more activated phenotype among TILs. In the ongoing first-in-human trial (NCT05263180), EMB-09 has already shown consistent pharmacodynamic modulation and preliminary antitumor signals, supporting its clinical translational potential (187).

In addition, the CTLA-4/OX40 bispecific antibody ATOR-1015 integrates dual immune checkpoint blockade and costimulation to achieve TME-directed activation. Comprising an optimized human CD86 IgV domain fused to an OX40 agonistic antibody, ATOR-1015 simultaneously blocks CTLA-4 and activates OX40 within the tumor milieu, thereby reducing intratumoral Treg frequencies and enhancing effector T-cell function. Across multiple syngeneic tumor models, ATOR-1015 significantly inhibited tumor growth, prolonged survival, and induced durable immunological memory, while also potentiating responses to anti-PD-1 therapy. Its preferential accumulation within tumors may mitigate systemic toxicity associated with conventional CTLA-4 antibodies, and it is currently under clinical investigation (NCT03782467) (188). **Table 5** summarizes the structural configurations and mechanistic principles underlying currently described OX40 bispecific antibodies.

**Table 5:** Structural and mechanistic features of OX40 bispecific antibodies.

BsAb name	Target pairing	Structural features/FcγR dependency	Key mechanisms	Major preclinical findings	Clinical status
FS120 (Fc-silent human IgG1)	OX40/CD137	Fc-silent, dual-agonist, FcγR-independent	Synergistic activation of CD4^+^/CD8^+^ T cells; reduced hepatotoxicity	Strong antitumor activity in multiple syngeneic models	Ongoing clinical development
PD-L1/OX40 BsAb(recombinant IgG2 bispecific antibody)	OX40/PD-L1	Tetravalent; PD-L1 VHH-mediated OX40 clustering	PD-L1–dependent OX40 clustering and costimulation	Enhanced T-cell activation; superior to monotherapy/combination	Preclinical
EMB-09(Fc-silent human IgG1)	OX40/PD-L1	Fc-silent; tetravalent	PD-L1–dependent OX40 agonism + PD-L1 blockade	Promotes effector-memory and stem-like CD8^+^ T cells	Phase I (NCT05263180)
ATOR-1015(human IgG1 with preserved Fc function)	OX40/CTLA-4	IgG1 format, dual-function	CTLA-4 blockade + OX40 agonism; Treg depletion	Tumor growth inhibition, increased CD8/Treg ratio	In clinical testing (NCT03782467)

Collectively, OX40-directed BsAbs overcome key limitations of OX40 monotherapies by optimizing receptor clustering, enhancing TME-selective activation, and integrating multiple immunomodulatory pathways. Future development should focus on refining crosslinking mechanisms, improving tumor-localized accumulation and specificity, establishing TME-based biomarker strategies, and exploring rational combinations with PD-1/PD-L1 inhibitors, cancer vaccines, or metabolic modulators to fully unlock the therapeutic potential of the OX40 pathway in cancer immunotherapy.

## 6 OX40 cooperates with other co-stimulatory molecules

Co-stimulatory molecules include immunoglobulin or TNF superfamily members (e.g., OX40, 4-1BB, GITR) (189), which promote T-cell activation, proliferation, and survival (33, 190, 191). OX40 synergizes with other co-stimulatory molecules through: Timing complementarity with CD28: CD28 initiates early IL-2 production, while OX40 (appearing ~48h later) promotes survival via Bcl-xL/Bcl-2 upregulation (32); Functional complementarity with 4-1BB: Both activate the PI3K/AKT/NF-κB pathway to enhance anti-apoptotic protein expression, OX40 is more critical for CD4^+^ T-cell survival (33), while 4-1BB preferentially acts on CD8^+^ T cells (192); Synergy with CD27: Dual CD27-OX40 signaling cooperatively inhibits Th17 differentiation and promotes Treg accumulation (193). OX40–OX40L signaling enhances T cell function via NFATc1 (67) and survivin pathways (69). OX40-deficient mice (LCMV/influenza models) show impaired CD4^+^ responses but intact CD8^+^ responses, confirming OX40’s role in CD4^+^ T-cell immunity (89). In sum, OX40 coordinates activation, survival, and effector function via spatiotemporal synergy with CD28 and 4-1BB, supporting co-targeting strategies like OX40/CD28 dual antibodies.

## 7 Challenges and future directions

OX40 signaling plays a central role in the activation, expansion, and memory formation of Teffs, with receptor expression peaking 48–72 hours after antigen engagement. However, its effects are highly time-sensitive. Sustained or improperly timed OX40 stimulation leads to the upregulation of inhibitory receptors such as PD-1, TIM-3, and LAG-3, resulting in activation-induced T-cell exhaustion and diminished therapeutic durability (194). Optimizing the timing, frequency, and duration of OX40 engagement is therefore a major challenge for maximizing therapeutic benefit while avoiding negative feedback.

A second and equally critical challenge arises from the complex, bidirectional roles of OX40 in Tregs. Unlike Teff cells, which transiently upregulate OX40 following activation, Tregs particularly ti-Tregs—express persistently high levels of OX40 and exhibit a strong dependency on OX40 signaling for their stability, survival, and tissue adaptation. Multi-omics studies demonstrate that ti-Tregs are transcriptionally, epigenetically, and metabolically distinct from peripheral Tregs, displaying heightened activation and tissue-adaptation signatures shaped by the TME. These findings imply that OX40 may regulate peripheral Tregs and ti-Tregs in fundamentally different ways. Yet, most mechanistic studies still rely on splenic or *in vitro*–generated Tregs, which may not accurately recapitulate ti-Treg biology. Key unresolved questions include whether OX40 signaling primarily destabilizes ti-Tregs by inducing Foxp3 downregulation, or whether it enhances their fitness under tumor conditions. Developing spatially and cell-type–selective approaches to selectively dismantle ti-Treg–mediated immunosuppression while preserving peripheral tolerance will be essential for improving OX40-based immunotherapy.

Beyond T-cell–intrinsic factors, therapeutic efficacy is profoundly influenced by the biophysical properties of OX40 agonists—particularly antibody isotype and Fcγ receptor engagement, which govern receptor clustering. First-generation OX40 monoclonal antibodies, predominantly IgG1, rely heavily on FcγR-mediated cross-linking to generate sufficient receptor clustering. This mechanism can preferentially deplete OX40^high ti-Tregs through antibody-dependent cellular cytotoxicity, thereby improving the Teff/Treg ratio within tumors. However, in tissues with low FcγR availability, these antibodies may fail to deliver adequate agonistic signals, contributing to inconsistent clinical responses. IgG2 antibodies, with reduced FcγR affinity, favor agonism over depletion; Fc-silent or Fc-null antibodies (e.g., LALA-PG variants) require structural multivalency to achieve clustering independently of FcγR; and Fc-engineered antibodies allow fine-tuning between agonistic activity and Treg depletion. Consequently, next-generation OX40 therapeutics—including Fc-engineered formats, multivalent or hexavalent agonists (e.g., INBRX-106), non-ligand-blocking variants (e.g., BGB-A445), and bispecific or cytokine-fusion platforms—represent rational and promising strategies to optimize both potency and tumor selectivity.

Taken together, future progress in OX40-directed immunotherapy will require addressing several interconnected challenges: (1) optimizing the temporal dynamics of OX40 stimulation to avoid exhaustion; (2) dissecting and selectively modulating the divergent effects of OX40 on peripheral Tregs versus ti-Tregs; (3) leveraging antibody isotype selection, FcγR engineering, and multivalent or bispecific agonist design to achieve efficient receptor clustering and favorable Teff/Treg modulation within tumors; and (4) incorporating primary mechanistic evidence to strengthen biological and therapeutic insights.

Overcoming these challenges, especially through ti-Treg–focused strategies and next-generation agonist engineering—will be pivotal for enabling OX40 to fulfill its therapeutic potential in cancer immunotherapy.

## 8 Conclusion

Collectively, the accumulated evidence positions OX40 as a biologically compelling yet clinically underrealized immunotherapeutic target. At the mechanistic level, OX40 functions as a central integrator of T-cell costimulation, coordinating TRAF-dependent NF-κB activation, PI3K–AKT survival signaling, lineage-specific differentiation programs, and the dynamic balance between effector and regulatory T-cell subsets. These multilayered regulatory functions enable OX40 to potentiate CD4^+^ and CD8^+^ T-cell expansion, reinforce memory formation, destabilize intratumoral Treg suppression, and recondition the TME toward an antitumor state. However, the impressive robustness of these mechanisms in preclinical systems has not yet been translated into commensurate clinical efficacy.

The recurring gap between pharmacodynamic activation and modest tumor regression observed in monotherapy and combination trials highlights the intrinsic biological constraints that govern OX40 function in human tumors. Key barriers include the narrow and transient temporal window of OX40 expression following TCR activation, the preferential and often dominant expression of OX40 on Tregs within multiple tumor types, immunometabolic deprivation that restricts costimulatory signaling, and suboptimal FcγR-mediated receptor clustering that limits agonist potency *in vivo*. These insights collectively dispel the notion that simple receptor engagement is sufficient for therapeutic benefit; instead, OX40 agonism requires precise alignment with the temporal, spatial, and immunometabolic context of the TME.

Looking ahead, renewed translational progress will depend on the rational integration of mechanistic constraints into therapeutic design. Next-generation antibody engineering—encompassing multivalent scaffolds, Fc-silent or Fc-enhanced constructs, and bispecific antibodies targeting PD-L1, CD137, or CTLA-4—promises to overcome limitations in receptor crosslinking and TME selectivity. Combinatorial strategies that synchronize OX40 stimulation with checkpoint blockade, metabolic reprogramming, vaccination, radiotherapy, or locoregional immune priming may expand the pool of activatable effector T cells while attenuating suppressive pathways. Equally critical will be the development of biomarker frameworks capable of identifying patients with favorable OX40^+^CD4^+^ TIL profiles, controlled OX40^+^ Treg abundance, and immunologically permissive microenvironments.

Ultimately, realizing the full therapeutic potential of OX40 will require a multidimensional strategy that couples mechanistic fidelity with clinical precision. By embedding immune kinetics, spatial distribution, Fc-engineering principles, and TME biology into the next phase of drug development, OX40-based therapeutics may evolve from an immunologic amplifier with inconsistent clinical activity into a foundational component of rational, multi-pathway cancer immunotherapy.
